# Efficient Chain Formation of Magnetic Particles in Elastomers with Limited Space

**DOI:** 10.3390/polym12020290

**Published:** 2020-02-01

**Authors:** Shota Akama, Yusuke Kobayashi, Mika Kawai, Tetsu Mitsumata

**Affiliations:** 1 Graduate School of Science and Technology, Niigata University, Niigata 950-2181, Japan; aaa.rock@icloud.com (S.A.); f19b131j@mail.cc.niigata-u.ac.jp (Y.K.); mikagoro@eng.niigata-u.ac.jp (M.K.); 2 ALCA, Japan Science and Technology Agency, Tokyo 102-0076, Japan

**Keywords:** soft material, stimuli-responsive gel, magnetic elastomer, percolation

## Abstract

The magnetic response of the storage modulus for bimodal magnetic elastomers containing magnetic particles with a diameter of 7.0 μm and plastic beads with a diameter of 200 μm were investigated by varying the volume fraction of plastic beads up to 0.60 while keeping the volume fraction of the magnetic particles at 0.10. The storage modulus at 0 mT for monomodal magnetic elastomers was 1.4 × 10^4^ Pa, and it slightly increased with the volume fraction of plastic beads up to 0.6. The storage modulus at 500 mT for bimodal magnetic elastomers at volume fractions below 0.25 was constant, which was equal to that for the monomodal one (=7.9 × 10^4^ Pa). At volume fractions of 0.25–0.40, the storage modulus significantly increased with the volume fraction, showing a percolation behavior. At volume fractions of 0.40-0.60, the storage modulus was constant at 2.0 × 10^5^ Pa, independently of the volume fraction. These results indicate that the enhanced increase in the storage modulus was caused by the chain formation of the magnetic particles in vacancies made of plastic beads.

## 1. Introduction

Magnetic elastomers are a type of stimuli-responsive soft material [1,2,3,4,5] and its physical properties alter in response to magnetic fields. The magnetic response for a magnetic elastomer is in general drastic; therefore, the material attracts considerable attention as actuators in the next generation of materials [6,7,8]. Magnetic elastomers consist of polymeric matrices, such as polyurethane, and magnetic particles with nano or micron sizes in diameter. When a magnetic field is applied to a magnetic elastomer, the elasticity increases due to the chain structure formation (restructuring) of magnetic particles, which is called the magnetorheological (MR) effect. So far, we have investigated the MR effect for polyurethane-based magnetic elastomers and found that bimodal magnetic elastomers with magnetic and nonmagnetic particles exhibit a significant MR effect compared with monomodal magnetic elastomers [9,10,11,12,13].

**Figure 1:**
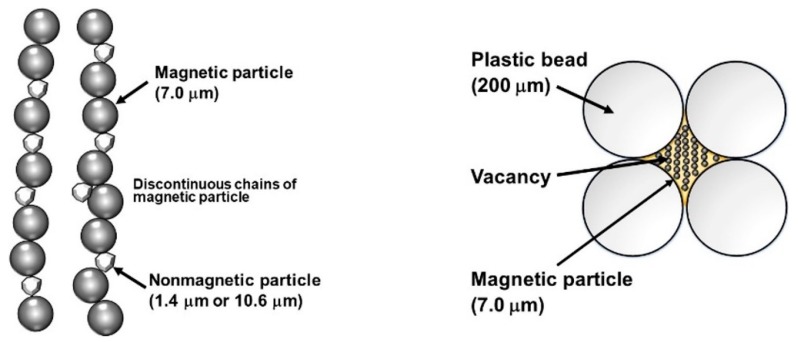
Schematic illustrations representing the mechanism for the magnetorheological (MR) effect for bimodal magnetic elastomers in our previous study and the scenario in the present study.

In our past studies, zinc oxide with a diameter of 10.6 μm or aluminum hydroxide with a diameter of 1.4 μm was used as nonmagnetic particles [9,11]. The increase in the storage modulus for bimodal magnetic elastomers containing zinc oxide particles of 12 vol.% was 500 kPa, which was achieved by applying a magnetic field of 500 mT, which is 4.2 times that used for the monomodal one (120 kPa) [9]. The increase in the storage modulus for bimodal magnetic elastomers containing aluminum hydroxide particles of 6.6 vol.% was 3.27 MPa, which is 4.3 times that used for the monomodal one (754 kPa) [11]. Figure 1 shows the schematic illustrations representing the mechanism for the MR effect for bimodal magnetic elastomers in our previous study and the scenario for that in the present study. In the previous study, the enhanced magnetorheology mentioned above is caused by bridging the discontinuous chains of magnetic particles with nonmagnetic particles. However, this mechanism is not efficient since a considerable amount of nonmagnetic particles is needed to raise the elasticity of magnetic elastomers.

In our present study, we proposed a new concept for the enhanced magnetorheology of bimodal magnetic elastomers. A large bead of poly(methyl-2-methylpropenoate) (PMMA) with a diameter of 200 μm was used as a non-magnetic particle. The diameter of the bead was large, and the interaction with the matrix of polyurethane was weak compared with nonmagnetic particles in past studies. Beads with a large diameter made vacancies in the magnetic elastomer, and magnetic particles were localized in the vacancies made of the plastic beads. The density of the plastic was low compared with inorganic compounds; therefore, the beads were not precipitated during the synthesis; this contributed to the random dispersion of vacancies in the elastomer. Bimodal magnetic elastomers with plastic beads also have advantages of transparency and weight saving. The density of zinc oxide and aluminum hydroxide is 5.8 and 2.4 g/cm^3^, respectively, meanwhile, the density of the plastic bead was 1.2 g/cm^3^. Magnetic elastomers with both a lightweight and huge magnetic response could be obtained. Bimodal magnetic elastomers with plastic beads have a possibility to transmit visible light, although the light may reflect at the interface between the matrix and plastic beads. This should be helpful for biologists or medical scientists who investigate the effect of substrate elasticity on the cell behavior using magnetic elastomers [14].

Here, we prepared bimodal magnetic elastomers with various volume fractions of plastic beads while keeping the volume fraction of magnetic particles constant, and discuss the addition effect of plastic beads on the magnetic response, the transmissibility of visible light, and weight saving.

## 2. Experimental Procedures

### 2.1. Synthesis of Magnetic Elastomers

Polypropylene glycols (P2000, G3000B, Adeka Co., Tokyo, Japan) with molecular weights of *M*_w_ = 2000 and 3000 were used for the matrix of magnetic elastomers. Tolyrene diisocyanate (Wako Pure Chemical Industries. Ltd., Osaka, Japan) and dioctyl phthalate (DOP, Wako Pure Chemical Industries. Ltd., Osaka, Japan) were used as a crosslinker and plasticizer, respectively. Carbonyl iron with a median diameter of 7.0 μm (CS Grade BASF SE., Ludwigshafen am Rhein, Germany) was used for magnetic particles. The saturation magnetization of carbonyl iron particles was 190 emu/g measured using a SQUID magnetometer (MPMS, Quantum Design Inc., San Diego, CA, USA). Plastic beads made of polymethylmethacrylate (PMMA) (Techpolymer, MBX-200, Sekisui Plastics Co., Ltd., Tokyo, Japan) with a mean diameter of 200 μm were used as nonmagnetic particles. Magnetic elastomers were synthesized using a prepolymer method. Polypropylene glycols are crosslinked with tolyrene diisocyanate. The concentration of the crosslinker remarkably affects not only the off-field modulus, but also the magnetorheological response. The molar ratio of –NCO to –OH group for the prepolymer was constant at 2.01 (=[NCO]/[OH]). Magnetic particles and plastic beads were mixed with prepolymer, linear polymer, plasticizer, and catalysis. The mixed liquid was poured into a silicon mold and cured for 30 min at 100 °C. DOP has a good chemical affinity with polypropylene glycols, which is a good solvent for crosslinked PPG elastomers. Similar to the crosslinker concentration, the concentration of the plasticizer strongly affects both the off-field modulus and the magnetorheological response. The weight concentration of DOP to the matrix without magnetic particles was fixed at 60 wt.%. The densities of the magnetic particles and plastic beads were 7.57 and 1.20 g/cm^3^, respectively. The volume fraction of magnetic particles was kept at 0.1; meanwhile, for plastic beads, it was varied from 0.10 to 0.60.

### 2.2. Dynamic Viscoelastic Measurement

Dynamic viscoelastic measurements were carried out for magnetic elastomers using a rheometer (MCR301, Anton Paar Pty. Ltd., Graz, Austria) at 20 °C. The strain was varied from 10^−5^ to 1 and the frequency was kept at 1 Hz. The sample was a disk that was 20 mm in diameter and 1.5 mm in thickness. The normal force initially applied to the magnetic elastomer was approximately 0.3 N.

### 2.3. SEM Observation

**Figure 2:**
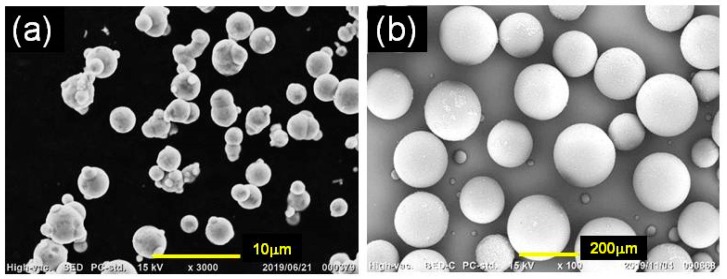
SEM photographs for (**a**) magnetic particles and (**b**) plastic beads (nonmagnetic particles).

The shape of the magnetic and nonmagnetic particles in the powder state and the particle morphology for monomodal and bimodal elastomers were observed using scanning electron microscopy (SEM, JCM-6000 Neoscope JEOL Ltd. Tokyo, Japan) with an accelerating voltage of 15 kV without a Au coating. SEM photographs for magnetic particles and plastic beads (nonmagnetic particles) are presented in Figure 2.

## 3. Results and Discussion

**Figure 3:**
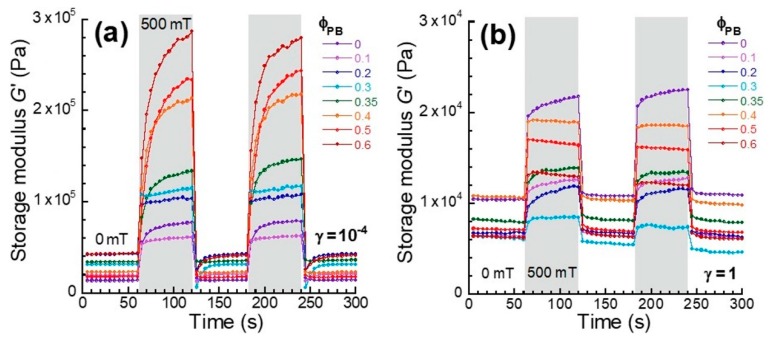
Magnetic field response of the storage modulus at strains of (**a**) 10^−4^ and (**b**) 1 for monomodal and bimodal magnetic elastomers containing plastic beads with various volume fractions.

Figure 3a exhibits the magnetic field response of the storage modulus at a strain of 10^−4^ (in the linear viscoelastic regime) for monomodal and bimodal magnetic elastomers containing plastic beads. A magnetic field of 500 mT was applied to the magnetic elastomers every 60 s. It was observed for all magnetic elastomers that the storage modulus was altered synchronously with the magnetic field, and completely recovered to the original modulus after removing the field. The storage modulus for bimodal magnetic elastomers at *ϕ*_PB_ < 0.3 rapidly increased due to the application of the magnetic field. This result coincided with our previous study showing that the alignment time improved by adding aluminum hydroxide nonmagnetic particles [11]. Meanwhile, at *ϕ*_PB_ > 0.35, the storage modulus gradually increased with time and it was not saturated within 60 s. It is considered that only magnetic particles move and form a chain structure at *ϕ*_PB_ < 0.3. At *ϕ*_PB_ > 0.35, large plastic beads moved, accompanying the movement of the magnetic particles, resulting in long relaxation time.

Figure 3b demonstrates the magnetic-field response of the storage modulus at a strain of 1 (in the non-linear viscoelastic regime) for monomodal and bimodal magnetic elastomers containing plastic beads. Similar to the results at low strain, all magnetic elastomers responded to the magnetic field at high strain. However, the time development of the storage modulus was opposite at high strain. A gradual increase of the storage modulus with time was observed for monomodal and bimodal magnetic elastomers with *ϕ*_PB_ < 0.3, and a rapid increase of the storage modulus with time was observed for bimodal magnetic elastomers with *ϕ*_PB_ > 0.35.

**Figure 4:**
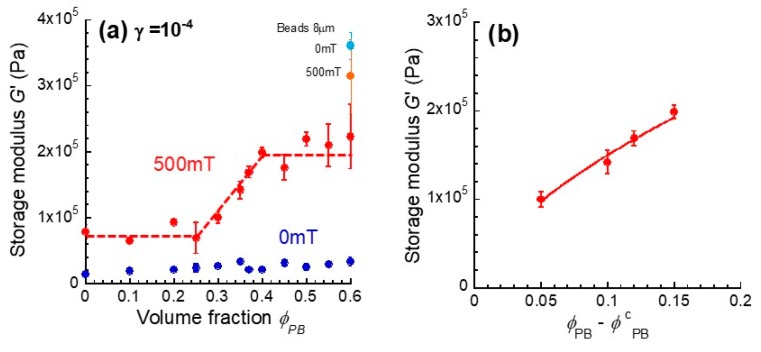
(**a**) Storage modulus at a strain of 10^−4^ at 0 and 500 mT as a function of the volume fraction of plastic beads and (**b**) storage modulus as a function of *ϕ*_PB_ − *ϕ*^c^_PB_ for monomodal and bimodal magnetic elastomers.

Figure 4a depicts the storage modulus at 10^−4^ for monomodal and bimodal magnetic elastomers as a function of the volume fraction of plastic beads. The storage modulus at 0 mT was almost constant, although the volume fraction of the plastic beads increased. In general, when the volume fraction of fillers was high, the storage modulus for the composite elastomers was higher than that for the matrix. The low storage modulus seen at *ϕ*_PB_ = 0.60 indicated that the plastic beads were randomly dispersed in the elastomer without direct contact between the beads. Of course, the interfacial effect between the matrix and plastic beads with a large diameter should be negligibly small compared with the small particles being several microns in size. Actually, when a plastic bead with a diameter of 8 μm was used, the storage modulus at 0 mT for the bimodal magnetic elastomer at *ϕ*_PB_ = 0.60 was 3.6 × 10^5^ Pa, which was far higher than that for the elastomer with large particles. In addition, the change in the storage modulus was very small and negative. This strongly indicated that the total interfacial area was dominant for the storage modulus of magnetic elastomers, i.e., the storage modulus could be raised by increasing the interfacial area, even though the interfacial interaction between the matrix and plastic beads was weak. The storage modulus at 500 mT for bimodal magnetic elastomers was equal to that of the monomodal one, and it was almost independent of the volume fraction at *ϕ*_PB_ < 0.25. This behavior was unusual, and it indicated that bridging did not occur between discontinuous chains of magnetic particles via plastic beads, even though the volume fraction of plastic beads was considerably high. At 0.25 < *ϕ*_PB_ < 0.40, the storage modulus significantly increased with the volume fraction of the plastic beads. It was considered that the magnetic particles bridged the gap between the plastic beads. An interesting thing was that the significant increase in the storage modulus was only seen at 500 mT and did not appear at 0 mT. In addition, the influence of the matrix elasticity on the magnetorheological effect was investigated. The storage modulus for polyurethane elastomers with (*ϕ*_PB_=0.40) and without plastic beads was (3.8 ± 0.35) × 10^4^ Pa and (3.3 ± 0.34) × 10^4^ Pa, respectively.

**Figure 5:**
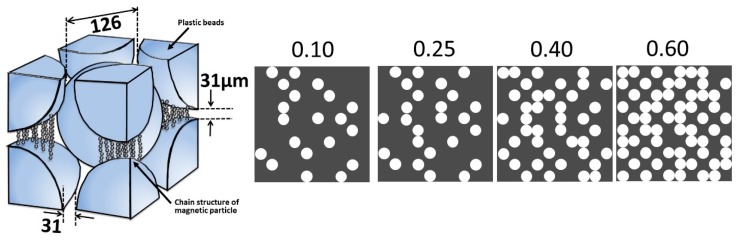
Schematic illustrations representing (**left**) the mechanism of the MR effect and (**right**) a realization of percolation behavior for bimodal magnetic elastomers with large plastic beads.

Therefore, an important factor for the stress transfer between plastic beads is the chain structure of magnetic particles, not the elasticity of the matrix. The onset volume fraction for the percolation is considered to be 0.25 < *ϕ*_PB_ < 0.30, which is close to the value of the cite percolation for a body-centered cubic structure (=0.248) [15,16]. Figure 4b shows the relation between the storage modulus and the volume fraction of plastic beads (*ϕ*_PB_ − *ϕ*^c^_PB_) for bimodal magnetic elastomers. The storage modulus at *ϕ*_PB_ > 0.25 obeyed a power low of *G*_500_~(*ϕ*_PB_ − *ϕ*^c^_PB_)^0.64^, although the critical exponent was relatively high compared with that of a 3D lattice (≈0.4) [15,16]. Accordingly, the plastic beads were packed in the structure of the body-centered cubic structure, and magnetic particles were filled in the vacancies between the beads. At *ϕ*_PB_ > 0.4, the storage modulus was not raised, although the volume fraction of plastic beads was increased. This indicated that the percolation possibility reached a maximum at *ϕ*_PB_ ≈ 0.5, which was 74% of the maximum packing ratio for the lattice of a body-centered cubic structure. It might be that the effective paths contributing to the storage modulus were not increased at *ϕ*_PB_ > 0.4 due to the increase in the number of branches of the percolated paths. Figure 5 exhibits the schematic illustrations representing the mechanism of the elasticity increase by the magnetic field for bimodal magnetic elastomers with plastic beads with a large diameter. The distance between the nearest neighbor beads was calculated to be 31 μm. Magnetic particles are forced to be localized in the vacancy and form a chain structure by the magnetic field, for example, a qualitative representation of the microstructure [17] and computed tomography images [18]. In our previous study, the storage modulus for bimodal magnetic elastomers containing small nonmagnetic particles (1.4 or 10.6 μm in diameter) gradually increased with the volume fraction of nonmagnetic particles, and a clear percolation threshold was not observed. This strongly indicated that the chains of magnetic particles were gradually connected via nonmagnetic particles with increases in the volume fraction of nonmagnetic particles [9,10,11]. The mechanism presented here was completely different from that observed in the past. Figure 5 also shows a realization of percolation behavior for bimodal magnetic elastomers with plastic beads. Each white circle represents a plastic bead, and the black part represents the magnetic elastomer, i.e., polyurethane and magnetic particles. The percolation of the stress between the plastic beads occurred via chains of magnetic particles at *ϕ*_PB_ = 0.25. The percolation path increased with the volume fraction of plastic beads at 0.25 < *ϕ*_PB_ < 0.40. At 0.40 < *ϕ*_PB_ < 0.60, both the percolation path and the branch of the paths increased with the volume fraction. 

**Figure 6:**
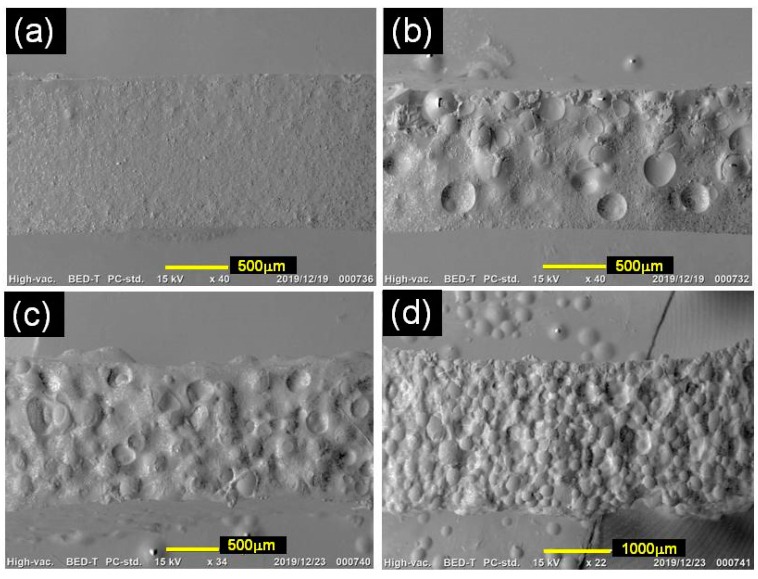

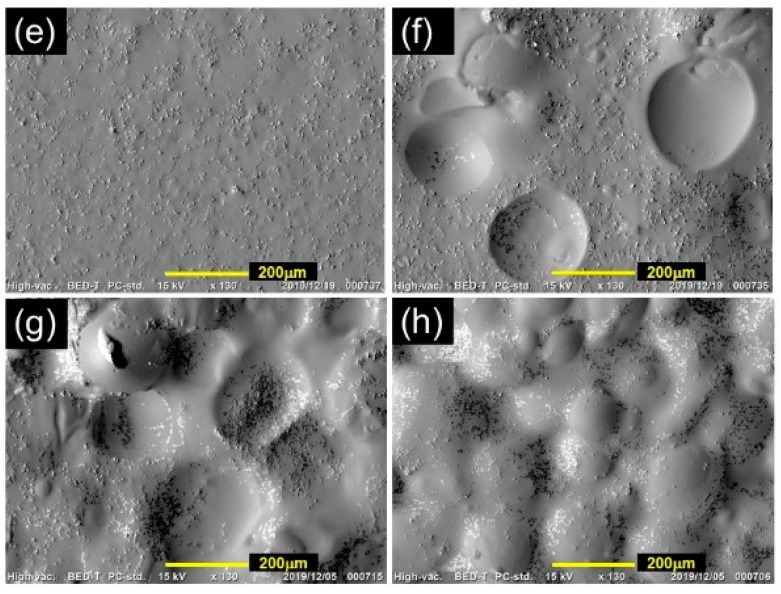
SEM photographs for (**a**,**e**) monomodal magnetic elastomers, (**b**–**d**,**f**–**h**) bimodal magnetic elastomers with various volume fractions of plastic beads (**b**,**f**) 0.25, (**c**,**g**) 0.40, and (**d**,**h**) 0.60.

Figure 6a,e indicates the SEM photographs for monomodal magnetic elastomers taken at different magnifications. Figure 6e demonstrates the fact that no clear aggregations of magnetic particles were found in the polyurethane matrix. However, the photo showed that there were two regions with different concentrations of magnetic particles, i.e., densely and sparsely dispersed parts. This result coincided with the previous results showing that carbonyl iron particles form secondary particles within the polyurethane matrix when ultrasonication is not carried out [19]. Figure 6c,g displays the SEM photographs for bimodal magnetic elastomers with plastic beads above the percolation threshold (*ϕ*_PB_ = 0.4). It was clear that magnetic particles were localized in the vacancy made by plastic beads, meaning the success of our scenario given that magnetic particles were gathered in a limited space. The hole made by removing the plastic bead showed a very smooth surface, suggesting that the interaction between the plastic beads and the matrix was weak (see Figure 6f). Figure 6d,h shows the SEM photographs for bimodal magnetic elastomers containing plastic beads with the maximum volume fraction (*ϕ*_PB_ = 0.60). It was clearly observed that magnetic particles were distributed in the vacancy.

**Figure 7:**
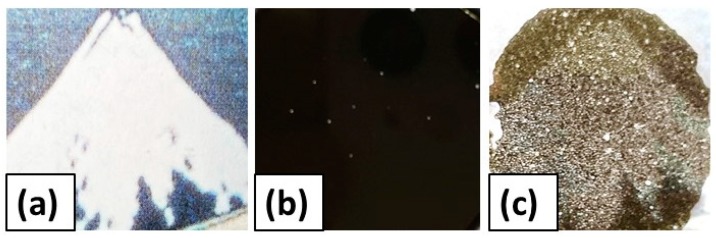

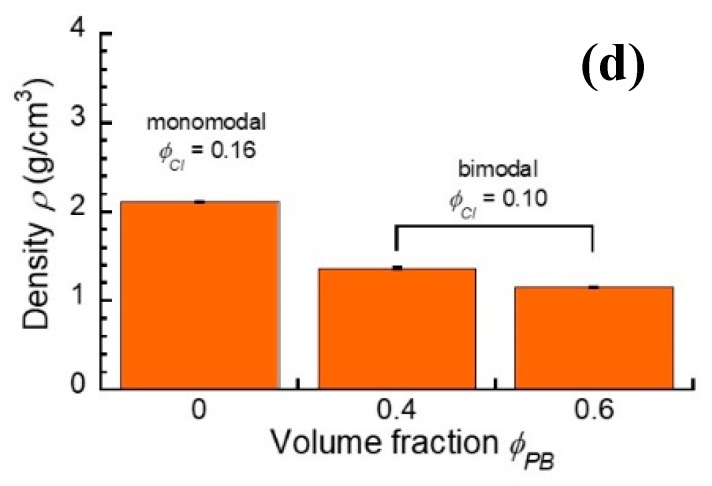
Photographs representing the transparency for monomodal and bimodal magnetic elastomers: (**a**) original paint, and (**b**) monomodal, and (**c**) bimodal magnetic elastomers were put on the original paint. (**d**) The density for monomodal and bimodal magnetic elastomers with plastic beads of *ϕ*_PB_ ≈ 0.4 and 0.6.

Figure 7 demonstrates the photographs representing the transparency and the density for bimodal magnetic elastomers with plastic beads obtained in this study. The transparency for bimodal magnetic elastomers with plastic beads (*ϕ*_PB_~0.60) was compared with that for monomodal ones. The increase in the storage modulus for the bimodal magnetic elastomer (=(8.00 ± 0.53) × 10^4^ Pa) was equal to that for monomodal one (=(7.1 ± 0.26) × 10^4^ Pa). In this experiment, the volume fraction of the magnetic particles was kept at 0.07 because the difference in the transparency for these elastomers was not clear to see. The thickness of the monomodal and bimodal magnetic elastomers was approximately 180 and 290 μm, respectively. It was found that the paint, the mountain with snow, could only be seen through the bimodal magnetic elastomer. The density for the bimodal magnetic elastomers with plastic beads (*ϕ*_PB_ ≈ 0.4, 0.6) was also compared with that for a monomodal one. The increase in the storage modulus was (2.7 ± 0.99) × 10^5^ Pa for monomodal, (1.8 ± 0.01) × 10^5^ Pa for bimodal with *ϕ*_PB_ ≈ 0.4 and (1.9 ± 4.2) × 10^5^ Pa for bimodal with *ϕ*_PB_ ≈ 0.6. The density was 2.11 g/cm^3^ for monomodal, 1.37 g/cm^3^ for bimodal with *ϕ*_PB_ ≈ 0.4 and 1.15 g/cm^3^ for bimodal with *ϕ*_PB_ ≈ 0.6. Weight saving of −59% was achieved by creating the limited space in the magnetic elastomer. It was the case that the particle distribution or the local rearrangement of magnetic particles [20,21] was considered and designed for an efficient MR effect in the vacancy. 

## 4. Conclusions

The magnetorheological response for bimodal magnetic elastomers with large plastic beads was investigated. It was found that the increment in the storage modulus for the bimodal magnetic elastomers was higher than twice that of the monomodal one. It could be considered that the enhanced magnetorheology was caused by an efficient chain formation of magnetic particles in a vacancy made by the plastic beads that packed into the body-centered cubic structure. In addition, it was found that the bimodal magnetic elastomer could partially transmit natural light, although the clarity was very low. At approximately half the weight of conventional elastomers, the bimodal magnetic elastomers showed the same level of elasticity changes. We believe that the magnetorheological features presented here are useful not only for applications but also for designing materials for new magnetorheological elastomers with a high efficiency and multiple functions.
